# Supplementary material for: Dietary protection against the visual and motor deficits induced by experimental autoimmune encephalomyelitis
Source: Front Neurol. 2023 Mar 2;14:1113954. doi: 10.3389/fneur.2023.1113954 (PMC10017782; doi:10.3389/fneur.2023.1113954)
Supplement: Supplemental Figure 1 — Diet compositions and weight changes. (A) Complete ingredients of the custom-formulated purified KD and CD diets. (B) Repeated measures graph showing patterns of weight changes in mice fed the KD or CD in the absence of EAE. For each individual mouse (n = 5/diet), weights are graphed at baseline (circles) and 5 weeks post-feeding of the indicated diet (squares). (C) Repeated measures graphs comparing baseline and final weights of female (left graph) and male (right graph) mice consuming a KD or CD during EAE. Weight comparisons span from the day of immunization (0 dpi; circles) to the termination of experiment (21 dpi; squares). (D) Shown are tabulated average glucose and ketone readings taken at baseline, 0 dpi, and 21 dpi, along with standard deviations (SD) and p-values. Statistical comparisons of KD vs. CD for females (F) and males (M) done by unpaired T-test. For (B–D), *p < 0.05, **p < 0.01, ****p < 0.0001. [file Data_Sheet_1.PDF]

A

	KD	CD
Ingredient	(g/kg)	(g/kg)
Casein	300	210
L-cystine	2.86	3.0
Corn starch		369.29
Maltodextrin		100.0
Sucrose		200.0
Cellulose	245.31	40.0
Medium Chain Triglycerides (MCT) oil	270.0	
Flaxseed oil	70.0	21.0
Canola oil	60.0	19.0
Mineral Mix, Ca-P deficient (79055)	18.5	13.4
Calcium Phosphate, dibasic	8.5	7.0
Calcium Carbonate	10.75	7.3
Vitamin Mix, Teklad (40060)	14.0	10.0
Ethoxyquin, antioxidant	0.08	0.01

B

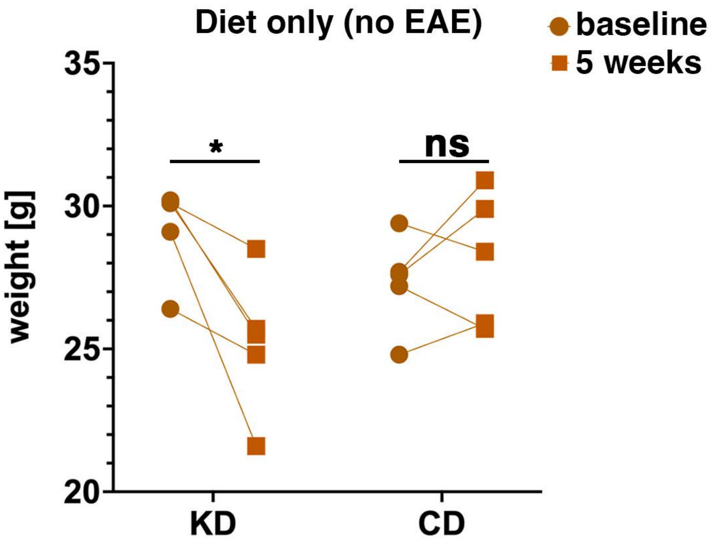

C

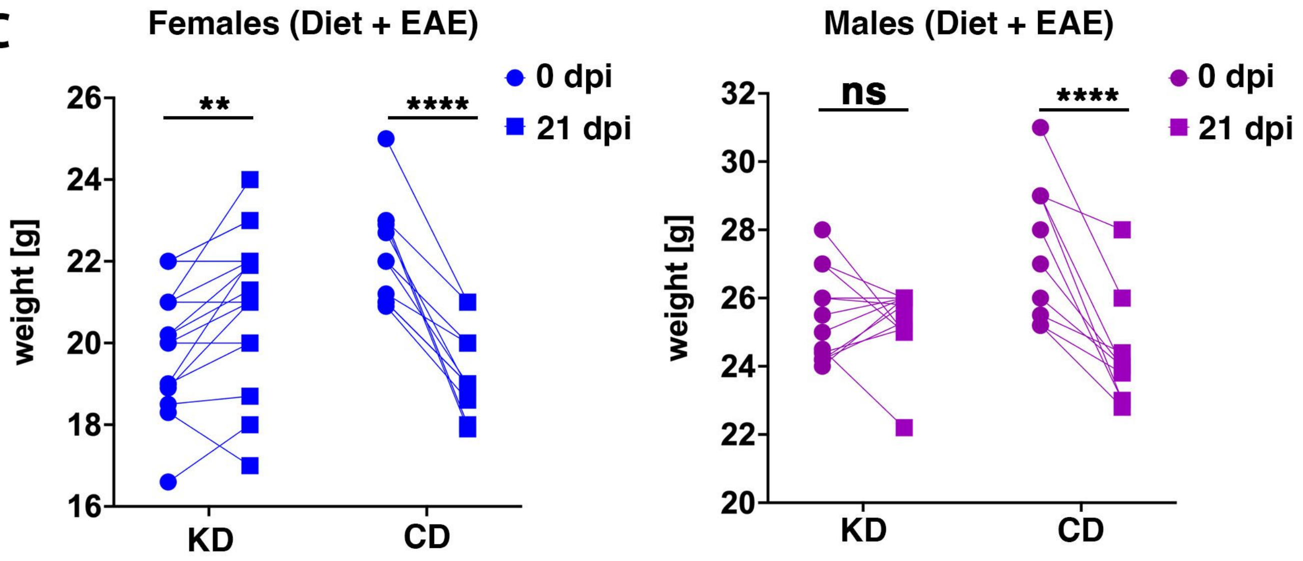

D

Time	group	Glu Avg (mg/dl)	Glu SD (mg/dl)	P val (CD)	Ket Avg (mmol/L)	Ket SD (mmol/L)	P val (CD)
Baseline	F KD	131.64	28.14	0.607	0.29	0.08	0.322
Baseline	F CD	137.77	33.00		0.33	0.11	
Baseline	M KD	142.11	27.80	0.442	0.36	0.05	0.226
Baseline	M CD	151.50	19.92		0.33	0.05	
0 dpi 2 weeks fed	F KD	96.76	26.81	*0.014	1.29	0.58	****0.000
0 dpi 2 weeks fed	F CD	120.47	24.42		0.30	0.07	
0 dpi 2 weeks fed	M KD	120.42	25.34	***0.000	1.22	0.35	****0.000
0 dpi 2 weeks fed	M CD	168.80	27.44		0.27	0.07	
21 dpi	F KD	88.59	35.28	0.165	1.55	0.55	***0.001
21 dpi	F CD	111.40	54.53		0.95	0.25	
21 dpi	M KD	95.17	52.56	0.243	1.52	0.38	****0.000
21 dpi	M CD	128.50	76.93		0.87	0.08	

Supplemental Figure 1

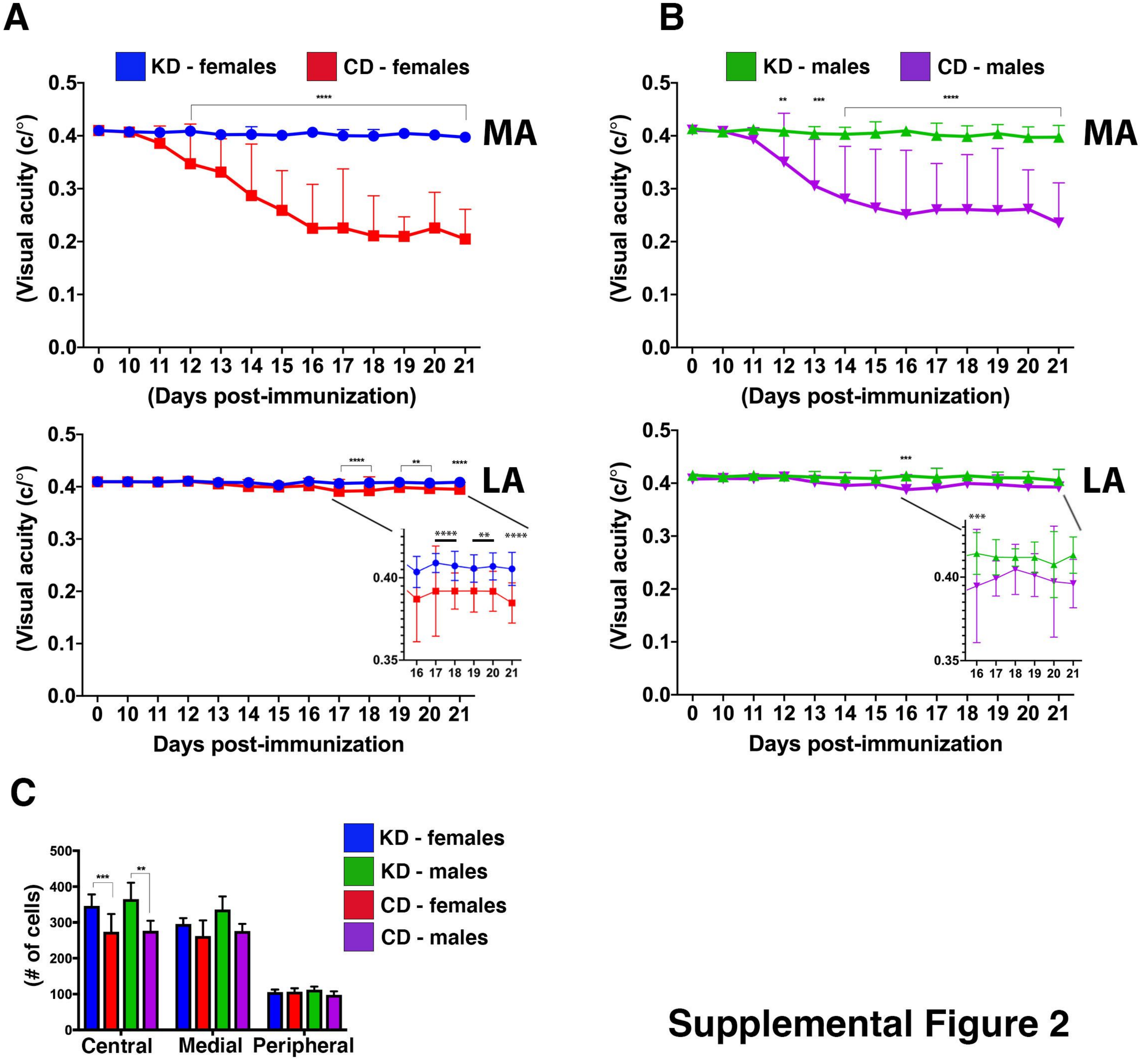

Supplemental Figure 2

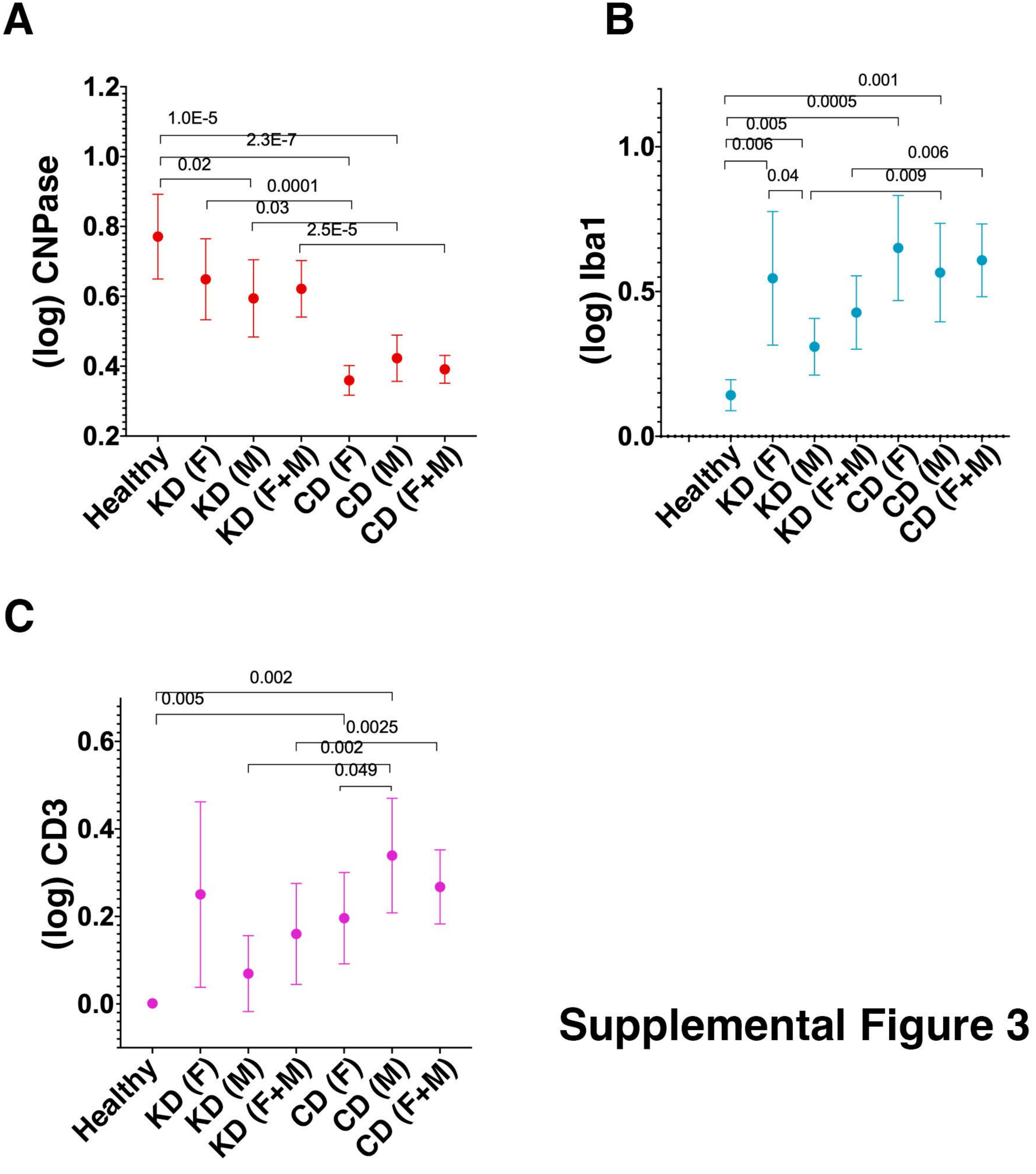

Supplemental Figure 3

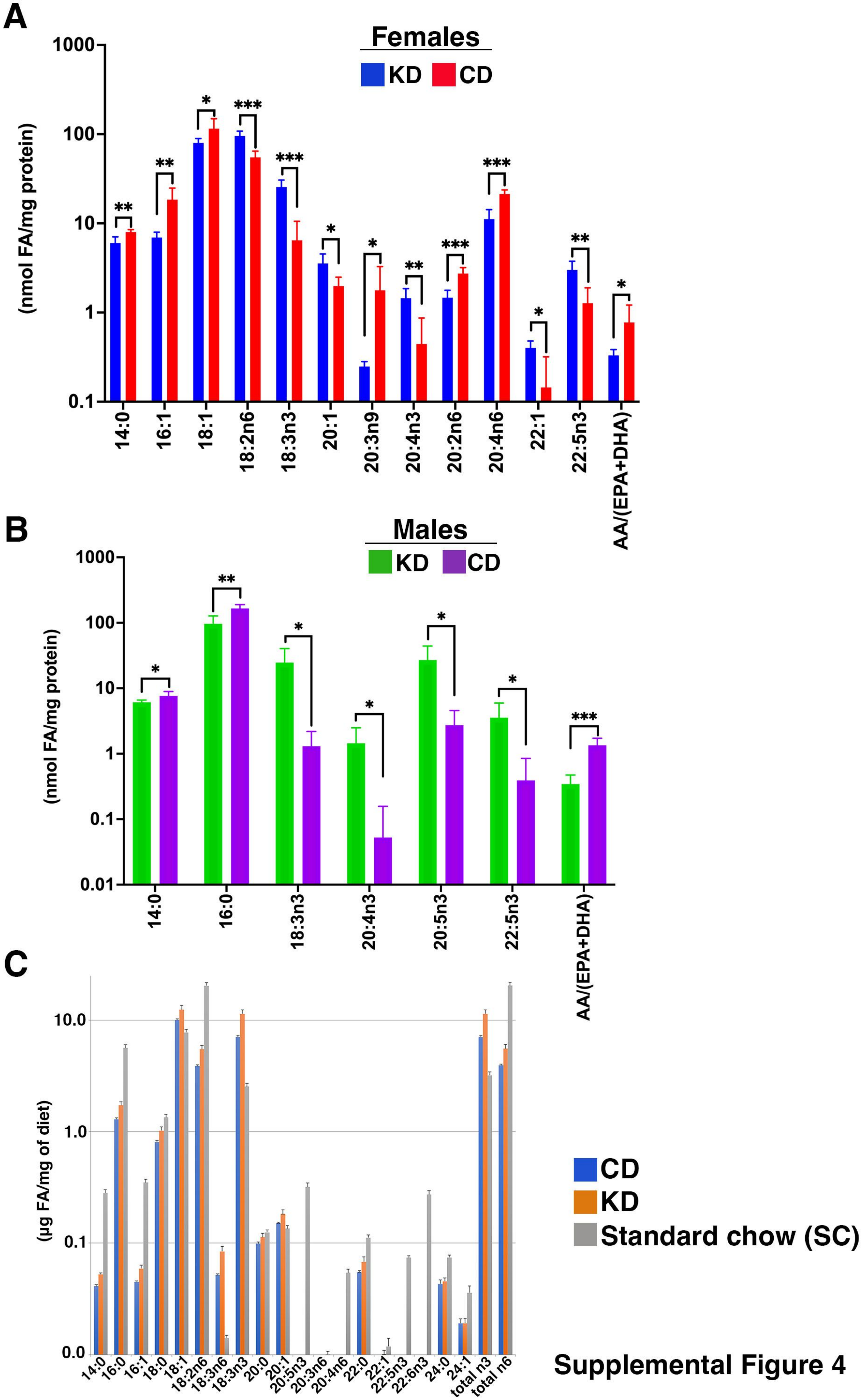

Supplemental Figure 4

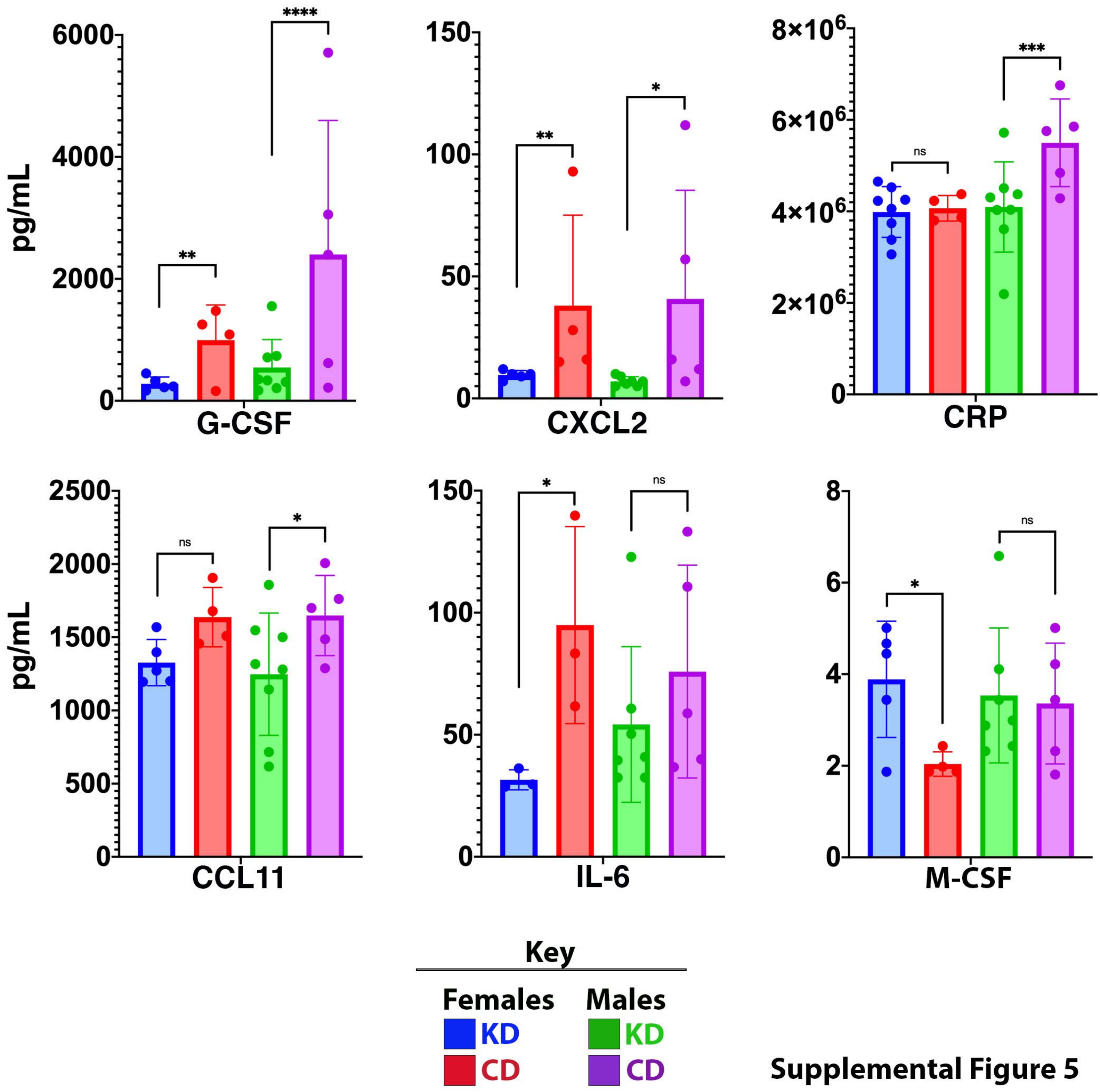

Supplemental Figure 5

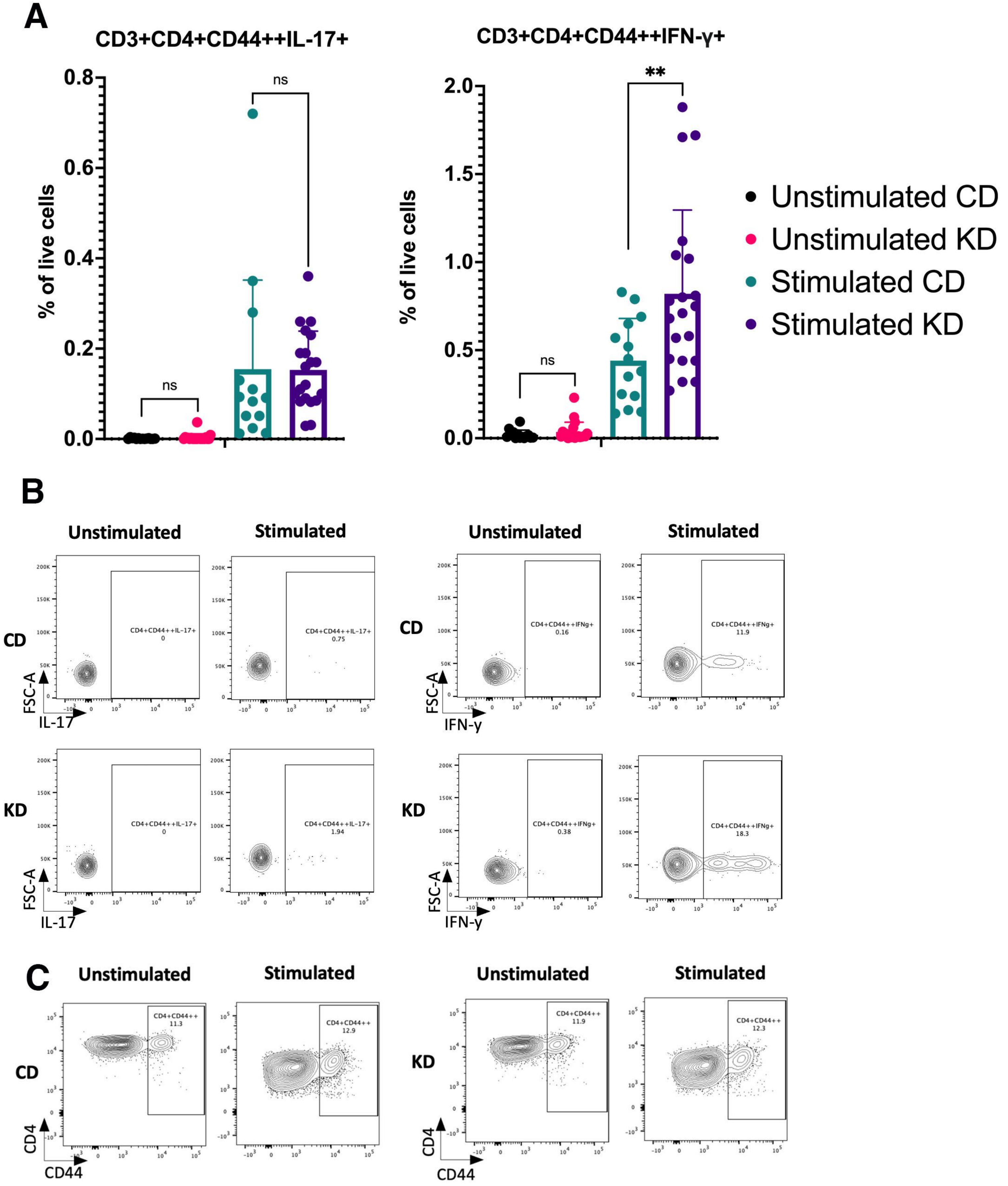

Supplemental Figure 6

A

Sex	# of immunized mice	Breakthrough KD-fed mice by 21 dpi	Percent
Females	32	3	9.38%
Males	26	2	7.69%
Total	58	5	8.62%

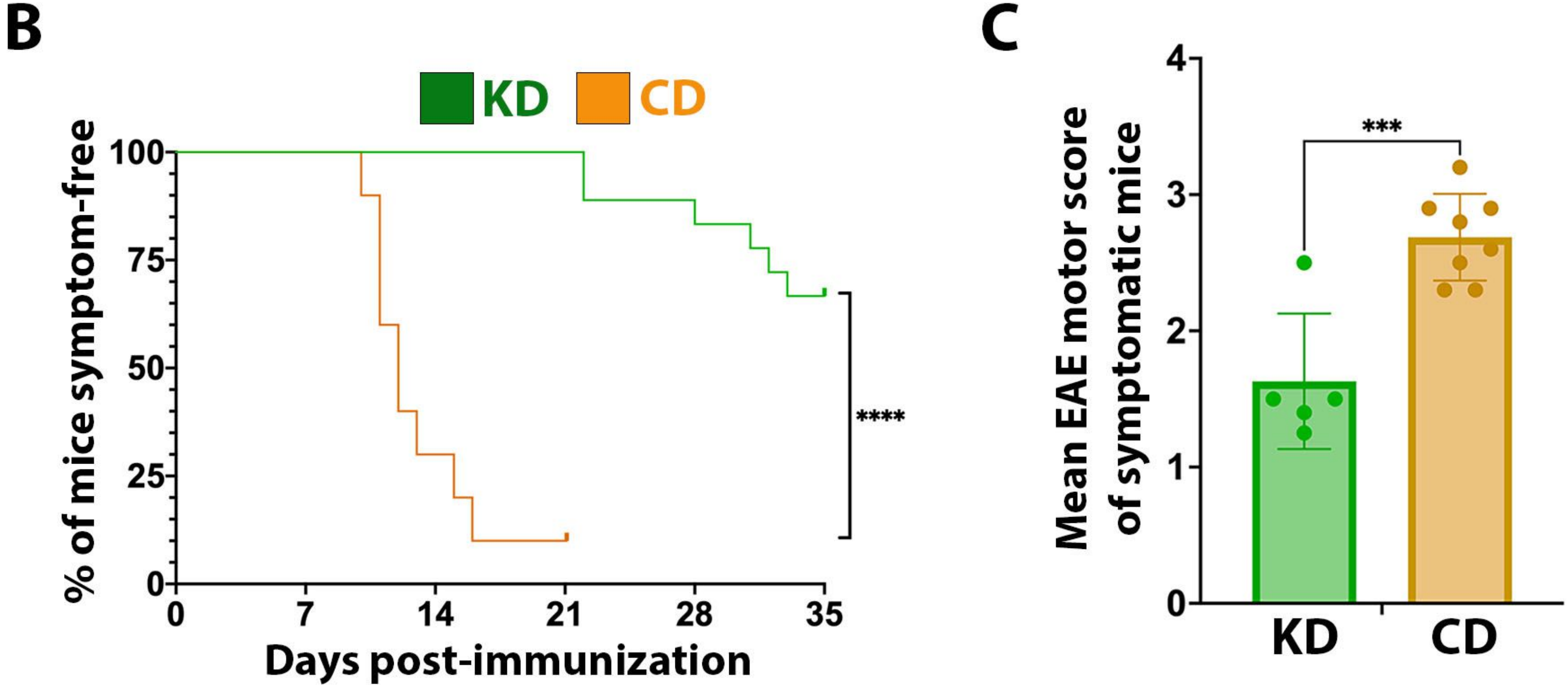

Supplemental Figure 7
